# Distemper in Dogs

**Published:** 1895-05

**Authors:** W. H. Martenet


### DISTEMPER IN DOGS.

BY W. H. MARTENET, D.V.S.

CANINE distemper is a disease prevailing all over the world, is of an infectious and contagious type, and extends to a few other species of animals, but is more commonly seen in the canine. It has no predilection for age, but is of a more virulent character in younger dogs, most likely on account of the inability of the undeveloped animal to understand the weakening effects of its attack. It is more enzootic in its character than otherwise, although it at times assumes an epizootic nature, and then is more virulent in its effects. This disease will admit of discussion at this time, when dogs have a high commercial value, and since so much loss is felt by its inroads on the kennels of breeders. The tendency at present is toward the raising of well-bred dogs, not only on account of their commercial value, but because this animal is capable of being a good friend to man, possessing a rare degree of intelligence for a brute and susceptible to man's friendship as no other animal is.

Dog shows during the winter months are the proper things, and at them congregate canines of many breeds from many States, and many of the animals represent to the owners goodly sums of money, and often realize for them either in stud fees or sales amounts that would seem great to the non-lover of dogs. At these shows, as happens at all live stock exhibitions, animals are shown that come from infected sources, and the result of their visit is the spread of the diseases incident to the species; thus it is that infection is spread, and thus it will ever be in all such shows unless a strict system of inspection is adopted that will preclude to the greatest extent the admittance of infected animals to the bench or stall.

Distemper is invidious in its advances, and often the source of infection is obscure, but if proper search is made, it can usually be ascertained. There is in my mind no doubt as to the disease emanating from an infection and never arising spontaneously. An incident in my practice tends to prove this assertion: A Chesapeake dog, five years old, was taken with all the symptoms of distemper, and from inquiry I learned that this animal but rarely went on the streets and had not for a long time prior to the time of the attack, yet he now had a serious attack of the disease. The lady owner, on racking her brain for some cause for the infection, remembered that there had been a disreputable-looking cat, with mattery eyes and of melancholy mien, roaming about the yard of the premises sometime before, thus explaining the source of the infection for her dog.

This disease is peculiar in that it has numerous complications and sequelæ, and simulates, as is asserted by some authorities, measles in man; and there is no doubt that in the course of time the analogy will be traced between it and some disease in man. First a bronchial affection is noted, that may run on, involving the lungs to a greater extent. Intestinal disturbances are a frequent complication, and may pass away readily or continue to a serious end. Nervous complications are also frequent, and often persist for a long time after the animal has otherwise recovered from an attack of the disease. The nervous complications may be acute inflammatory conditions of the cord or brain, showing themselves during the activity of the attack, or they may be sequelæ in the way of an acute or chronic chorea, or partial or total paralysis of one or more members, principally, however, the hind legs. Choreaic movements are noted following attacks of distemper, and often continue during the life of the animal, being seen more frequently in the legs, sometimes in the head and neck, rarely in the jaws. The eye is intimately involved in distemper; in the great majority of cases conjunctivitis is seen, but occasionally we may meet with a case in which the eyes escape, then the attack is extremely mild. The eye trouble may remain benign; more often, however, it increases in seriousness, affecting the cornea mainly. I have a case in hand in which the sequel has been an inflammation of the optic nerves, resulting in a nearly total loss of vision, and which is improving, but slowly, under stiff doses of strychnia. These eyes had the appearance at first of a simple ophthalmia of rather severe type, but after the application of atropia and boric acid wash for three or four times the lids were carried widely opened, exposing a highly-dilated iris that fooled me for several days. After the dilatation continued for four or five days, however, I noted the trouble, and by consent of the owner called in an oculist, who bore out my diagnosis, and advised more heroic doses of strychnia than the dog was being given by me. Improvement is but slowly taking place, and the prognosis looks unfavorable.

After a dog has contracted distemper it is usually an easy matter to diagnose the trouble.

A dulness is first seen that is unusual, anorexia, staring coat, redness of the conjunctiva, disposition to lie in cool or retired spots, shivering, followed by feverishness, much thirst, scanty urine, constipation or diarrhœa, and a cough that looks more like efforts at vomiting than a cough proper and that is paroxysmal in nature. Sometimes an eruption is seen on the belly and thin-skinned parts of the legs in the shape of small elevations that are well defined and that proceed to vesication, then are covered by scab. These vesicles are not always present. These symptoms follow a stage of incubation that is obscure and exhibiting symptoms that can be relied upon.

After the disease has well started the cough increases and is accompanied by retching and gagging that sometimes cause the expulsion of a clear viscid fluid principally composed of saliva; sometimes of a greenish matter, consisting mainly of bile when the disease is complicated with hepatic disturbances; but the stomach is usually emptied of its contents during these paroxysms. The anorexia persists and causes emaciation; the thirst is intense, and from symptoms shown the fever is evidently great. In young dogs at this time epileptic convulsions are often present, denoting the participation of the brain in the effects of the disease; and this is always an unfavorable condition of affairs that will seldom respond to treatment and generally ends in the death of the patient. The eyes may now exhibit a discharge of a yellowish, gluey matter, accompanied by an opacity of the cornea, denoting the presence of keratitis. There is also a copious discharge from the nostrils of an at first clear, then turbid, sticky mucus that continues for quite a length of time. These symptoms may gradually disappear under treatment, or they may increase in intensity, and then we see a rapid loss of flesh, with great weakness; the discharge from the eyes and nose is great, and the odor is very disagreeable; the diarrhœa, which is always seen in this condition of the disease, is copious and also extremely fetid in character, and may have streaks of blood in it; the prostration is great, and the animal can move about only with great difficulty; the symptoms now increase in severity and continue until death ensues from exhaustion. If nervous symptoms have developed, the convulsions are frequent, being accompanied by moans and sharp cries that are of unusual sound, denoting serious involvement of the brain, and continue until a state of coma is reached, when death shortly ensues. If the eyes have been specially affected the conjunctivitis is followed by a keratitis that may run on to ulceration of the cornea and loss of the contents of the members, or a chronic opacity of the cornea may result with consequent partial or total blindness. If the lungs have been the seat of the principal trouble, the bronchial irritation of the first few days may develop into bronchitis, involving the smaller bronchii and capillaries, and then broncho-pneumonia supervenes with a general breaking down of the lungs, resulting in death.

The treatment of distemper is varied, and especially so if one follows the course laid down in the numerous dog-books, whose number is legion, and whose value is equal that of the great number of horse-doctor works that are scattered so freely among the laity. Many advise on the start the administration of an emetic, followed by a cathartic to clean out the system of the animal, of the infection, probably. This, in my mind, is a very improper start, and one which, instead of relieving, tends to weaken the animal and lay it open to the debilitating effects of the disease, and also to start a beautiful case of diarrhœa that is hard to control. Distemper is a disease that runs a regular course, and nothing will cut it short, therefore the plan of treatment is to keep the patient in good hygienic condition and allay presented symptoms. There is great nausea, which is to be allieviated and overcome by calmatives, such as prepared chalk, sulphite of soda, hydrocyanic acid, lime-water, etc.

If constipation exists, leave it alone, and nature will take its course in this regard in a day or two, and even a diarrhœaic condition may ensue. If diarrhœa is present, however, treatment in this direction is indicated, accompanied by stomachics and tonics. The febrile phenomena are to be relieved by quinine, antifebrin, sweet spirit of nitre, etc. If such prostration is noticed, stimulants, preferably alcoholic (since ammoniacal stimulants are too severe on the sensitive mucous tracts), in the shape of port wine, sherry wine, or any of the light wines, or even whisky, may be given when prompt stimulation is needed.

When the bronchii and lungs are principally affected, anodynes, expectorants, etc., are to be used in connection with the stimulation of the chest with a strong lotion, and warm clothing with good ventilation of quarters provided. Nervous sequelæ are best treated by tonics, nux vomica or strychnia; general tonics acting well here, also, in restoring the general health, and with it the gradual improvement in the nervous symptoms ensues. The eye troubles are to be met with the customary washes, the milder the better, except when a serious aspect is noted, when more severe treatment is necessary. Nervous complications are treated mainly according to the condition exhibited; but usually demand nerve sedatives, principally the bromides.

The persistent refusal of food usually yields after a time to the stomachic and tonic treatment, and a gradual return of the appetite will take place. I have had dogs go without food absolutely for a solid week when suffering from distemper, and then make better recoveries than if they had been eating well during its course.

However, when much emaciation exists and the appetite is poor, broths and soups skimmed of the fats are often taken with relish. An egg beaten up with milk and liquor is very strengthening, and can be given with a spoon. The forcing of food down the animal's throat against its desire is not proper, and will not help the condition at all. It is my custom to advise the feeding of anything that the dog has an appetite for, but not in large quanties. One patient was very fond of smoked herring, the roe of which was the breakfast dish of the owner, and ate it while he would take nothing else. The appetite of the dog with distemper is very changeable, and should be humored. The eyes and nostrils are to be attended to daily by sponging and cleaning. A wash of borax—one drachm to the pint of lukewarm water—makes a good sponge wash for the eyes, and might be used to clean the nostrils also. The ancient use of tar in the kennel and on the patient is not to be deprecated, as the balsamic odor is beneficial to the irritated membranes, and, in my opinion, relieves the catarrhal condition of the mucous tracts. I advise the use of oil of tar to the nose, from the line of the eyes to the tip of the nose, believiug that the smell of the tar certainly is of virtue.

With all our efforts the case will often keep on the downward track, and cannot be turned, and will get away from us, so that at times there is not much satisfaction or honor to be obtained in our dog practice, yet the owner usually has a knowledge of the dangerous character of this disease, and if he has not it does not take long to impress him with the fact of it being extremely dangerous, so that, if perchance the canine should make a good recovery, we may make a good hit.

BALTIMORE, MD.

---

Little or no change has taken place in the tools of a horseshoer from those used in that trade three hundred years ago.

In a year the food eaten by a horse is estimated at nine times his weight, that of a cow nine times, that of an ox six times.

A recent case of diphtheria in one family was traced to a cat as the bearer of the same through its migrations from one house to the other.

Owls cannot move their eyes around, but to compensate for this they are able to turn their heads in almost a complete circle without a motion of their bodies.

The shark, it has been observed, manifests a distinct choice for people of certain races, and will eat an Asiatic in preference to a negro, and a European rather than either.

The eligible list for diplomas in the New York College of Veterinary Surgeons numbered fifty-three (53). Of these forty-two (42) came up for graduation, and thirty-one (31) were successful.

The West Chicago Street Railway Company owns in their horse called Jupiter one of the tallest and largest horses in this country—bay in color, bony and ungainly, nine years of age, of Kentucky parentage, weighing about 2200 pounds and stands twenty-one hands high.

Only three candidates presented themselves for examination on April 2d at the regular meeting of the State Board at Columbus, Ohio. Two were graduates, one of the Chicago Veterinary College and one of the Ontario Veterinary College, both passing; the third, a non-graduate, failed to attain the average required.

